# Validity and sensitivity of instrumented postural and gait assessment using low-cost devices in Parkinson’s disease

**DOI:** 10.1186/s12984-020-00770-7

**Published:** 2020-11-11

**Authors:** Ignacio Álvarez, Jorge Latorre, Miquel Aguilar, Pau Pastor, Roberto Llorens

**Affiliations:** 1 Fundació Docència i Recerca Mútua de Terrassa, Terrassa, Barcelona Spain; 2 Movement disorders Unit, Department of Neurology, Memory Disorders Unit, Hospital Universitari Mútua de Terrassa, Terrassa, Barcelona Spain; 3 Neurorehabilitation and Brain Research Group, Instituto Interuniversitario de Investigación en Bioingeniería, Universitat Politècnica de València, Ciudad Politécnica de la Innovación-Building 8B-Access M-Floor 0, Camino de Vera s/n, 46022 Valencia, Spain; 4 NEURORHB. Servicio de Neurorrehabilitación de Hospitales Vithas, Río Tajo 1, 46011 Valencia, Spain

**Keywords:** Parkinson’s disease, Gait, Posture, Kinect, Wii balance board

## Abstract

**Background:**

Accurate assessment of balance and gait is necessary to monitor the clinical progress of Parkinson’s disease (PD). Conventional clinical scales can be biased and have limited accuracy. Novel interactive devices are potentially useful to detect subtle posture or gait-related impairments.

**Methods:**

Posturographic and single and dual-task gait assessments were performed to 54 individuals with PD and 43 healthy controls with the Wii Balance Board and the Kinect v2 and the, respectively. Individuals with PD were also assessed with the Tinetti Performance Oriented Mobility Assessment, the Functional Gait Assessment and the 10-m Walking Test. The influence of demographic and clinical variables on the performance in the instrumented posturographic and gait tests, the sensitivity of these tests to the clinical condition and phenotypes, and their convergent validity with clinical scales were investigated.

**Results:**

Individuals with PD in H&Y I and I.5 stages showed similar performance to controls. The greatest differences in posture and gait were found between subjects in H&Y II.5 and H&Y I–I.5 stage, as well as controls. Dual-tasking enhanced the differences among all groups in gait parameters. Akinetic/rigid phenotype showed worse postural control and gait than other phenotypes. High significant correlations were found between the limits of stability and most of gait parameters with the clinical scales.

**Conclusions:**

Low-cost devices showed potential to objectively quantify posture and gait in established PD (H&Y ≥ II). Dual-tasking gait evaluation was more sensitive to detect differences among PD stages and compared to controls than free gait. Gait and posture were more impaired in akinetic/rigid PD.

## Background

Parkinson’s disease (PD) is a neurodegenerative disease, characterized by motor impairments with particular impacts on posture and gait, leading to postural instability and falls [1]. Previous PD research has described an overall decline in gait associated with neurodegeneration. Although gait does not appear to differ significantly between PD patients and healthy controls during the early stages of PD, including those categorized as Hoehn and Yahr (H&Y) I–II.5 [2], worsening gait speeds are expected across all H&Y stages and become especially evident in patients during the moderate and severe stages, including those categorized as H&Y ≥ II.5 [3]. In addition to an association with the time course of clinical symptoms, worsening spatiotemporal gait parameters have been identified in individuals with akinetic/rigid (AR)- or freezing-PD phenotypes [4, 5]. Research has also demonstrated that the performance of dual tasks has a significant and consistent negative effect on gait performance in PD patients, at different gait speeds and for different tasks [6], and dual-task associated gait evaluation can be used to distinguish individuals with de novo PD from healthy controls, which is not possible through the evaluation of regular walking [7]. Standardized clinical scales are commonly used to assess posture and gait in clinical settings, but their limited accuracy and intrinsic subjectivity may dramatically reduce their reliability [8]. To overcome these limitations, different instrumented tools, such as force plates and motion-tracking systems, have been developed for the accurate assessment of posture and gait, respectively [9, 10]. However, instrumented systems can be time- and space-consuming and are often unaffordable for most clinics, limiting their widespread use. Recent low-cost interactive technologies have shown comparable accuracy to laboratory-grade instrumented systems while being portable and affordable [11–13].

Wearable sensors that embed accelerometers and gyroscopes have been widely used to investigate posture and gait in subjects with PD [14, 15]. However, the accuracy of the assessment using wearable sensors depends on sensor location, sensor-to-segment alignment, and, frequently, on the number of sensors, which increases the total cost and obtrusiveness of the setting [16]. The Wii Balance Board (WBB) (Nintendo, Kyoto, Japan) and the Kinect for Windows v2, also known as Kinect v2 (Microsoft, Redmond, WA, USA), in contrast, potentially enable human motion tracking without the limitations of wearable sensors. The WBB is a portable and low-cost force platform that can estimate the center of pressure with comparable accuracy to that of laboratory-grade systems [17]. Preliminary research involving individuals with different neurological diseases, including PD, and different laboratory-grade systems has also shown that the WBB is a valid and feasible tool for the measurement of postural control and static balance during these conditions [18–20]. The low-cost, infrared camera Kinect v2 enables the motion tracking of the main joints of the human body, without requiring wearable markers. The potential for the Kinect v2 to determine the spatiotemporal and kinematic variables associated with gait has been also investigated in patients with neurological diseases, with promising results [21–25]. Research has also shown good agreement between the Kinect and a laboratory-grade three-dimensional motion analysis system during measurements of movements of individuals with PD [26]. However, existing studies examining individuals with PD have included limited numbers of participants [21, 22, 25], which limits the ability to extrapolate results, or have only investigated specific parameters, without performing comprehensive postural or gait assessments [21, 22, 25].

We hypothesized that the WBB and the Kinect v2 can be used as sensitive and valid tools for the identification of postural and gait disturbances, which are present at PD onset in most individuals. The aim of this study was to determine the sensitivity of these tools for the evaluation of impairments associated with the clinical condition and phenotype of PD patients, and their convergent validity with conventional clinical scales.

## Methods

### Participants

A convenient sample of individuals with PD were recruited from the Movement Disorders Unit of the Hospital Universitari Mútua de Terrassa (Terrassa, Barcelona, Spain), from January 2017 to November 2018. Individuals were included if they met the UK Brain Bank criteria for PD [27], were categorized on the modified H&Y scale as stages I–III [28], were able to walk 5 m without assistance or device, and did not present dyskinesia or significant cognitive impairments, as defined by scores > 18, 21, or 24 on the Mini-Mental State Examination, depending on the individual education level [29], at the time of evaluation.

Healthy participants, with no concurrent neurological or systemic disorders, postural or gait impairments, or lower limb prostheses, were primarily recruited among the relatives and caregivers of participants with PD.

Written informed consent was obtained from all participants, and the present study was approved by the Ethics Committee of the University Hospital Mútua de Terrassa.

### Instrumentation

Postural control was assessed using a WBB-based posturography application, which included a modified clinical test of sensory interaction on balance (mCTSIB) and a limits of stability (LOS) test [19]. The mCTSIB investigated alterations in the sensory integration and processing, as a measure of sway, by analyzing variations in the center of pressure while barefoot subjects remained as still as possible, in a standing position, for 30 s, under four different sensorial conditions: eyes opened and closed on the platform and eyes opened and closed on a piece of foam over the platform. The LOS test quantified the maximum displacement achievable for the center of pressure while maintaining contact between the soles of the feet and the platform, in eight directions, and the reaction time necessary to initiate the movement.

Spatiotemporal gait features were assessed with a Kinect v2-based gait analysis application [30], which required participants to walk towards the Kinect v2 at least three times, from a distance of 6 m, and estimated the most widely-used spatiotemporal and kinematic gait parameters; toe kinematics were not assessed using this application due to poor reliability [22, 31].

A WBB and a Kinect v2 were used to estimate the center of pressure of the participants and to retrieve the 3-dimensional body pose of participants, respectively. A conventional laptop was used to run both applications.

### Procedure

All participants with PD underwent a complete neurological evaluation, including the collection of demographic and clinical data, and were categorized by neurologists (MA, PP) into tremor dominant (TD), akinetic/rigid (AR) or mixed (MX) phenotypes as described previously [32, 33] (Table 1). Individuals with PD were also assessed using a battery of clinical scales, including the Unified Parkinson's Disease Rating Scale (UPDRS) [34], the Neuropsychiatric Inventory Questionnaire [35], the Non-Motor Symptoms Scale [36], the modified-Minnesota Impulsive Disorders Interview [37], the 39-Item Parkinson's Disease Questionnaire [38], the Tinetti Performance Oriented Mobility Assessment [8], the Functional Gait Assessment [8], and the 10-m Walking Test [39]. Finally, all participants, including those with PD and healthy subjects, were assessed by the instrumented posturography and gait analysis applications. For the dual-task condition, subjects were instructed to walk towards the camera while simultaneously counting backward from a number randomly picked between 60 and 110, either by ones, by twos (odd and even), by threes, or by sevens, according to the educational level and individual capabilities of each participant, which were assessed before the experiment. Subjects were allowed to rest between tests if needed.

**Table 1:** Demographic and clinical characteristics of the participants with Parkinson’s disease

	H&Y I-I.5	H&Y II	H&Y II.5
Number of participants (n)	11	37	6
Age (years)	68.0 ± 11.8	68.7 ± 10.2	74.5 ± 8.9
Sex (n, %)			
Women	2 (18.2%)	18 (48.6%)	4 (66.7%)
Men	9 (81.8%)	19 (51.4%)	2 (33.3%)
Height (cm)	170.2 ± 9.4	164.2 ± 6.2	162.2 ± 8.3
Weight (kg)	79.3 ± 15.4	72.1 ± 14.9	75.0 ± 11.7
Education level (years)	9.0 ± 5.7	9.0 ± 4.3	5.5 ± 3.2
Disease duration (years)	3.8 ± 2.1	6.7 ± 4.0	8.9 ± 5.6
Phenotype (n, %)			
Tremor dominant	6 (54.5%)	18 (48.6%)	3 (50.0%)
Akinetic/rigid	2 (18.2%)	6 (16.2%)	2 (33.3%)
Mixed	3 (27.3%)	13 (35.1%)	1 (16.7%)
Positive ioflupane I123 injection test (n)	5 out of 5 (100%)	12 out of 15 (80%)	2 out of 2 (100%)
Movement disorders*			
Tremor type			
Postural	5 (45.5%)	9 (24.3%)	1 (16.7%)
Rest	2 (18.2%)	6 (16.2%)	2 (33.3%)
Both	3 (27.3%)	19 (51.4%)	2 (33.3%)
Freezing	0	9 (24.3%)	1 (16.7%)
Festination	2 (18.2%)	8 (21.6%)	2 (33.3%)
Dyskinesia	0	6 (16.2%)	0
Fallers	1 (9.1%)	8 (21.6%)	2 (33.3%)
Schwab & England Activities of Daily Living Scale (100%) (n)	11 (100%)	24 (64.9%)	4 (66.7%)
REM Sleep Behavior Disorder (n)	4 (36.4%)	21 (56.8%)	1 (16.7%)
Unified Parkinson's Disease Rating Scale			
I. Mentation, Behavior And mood	1.4 ± 1.4	2.5 ± 2.3	1.8 ± 1.8
II. Activities of daily living	2.2 ± 1.4^2.3^	7.4 ± 4.2	7.3 ± 3.1
III. Motor examination	8.4 ± 4.0^2.3^	18.7 ± 7.5	16.8 ± 4.6
III. Motor examination (lower body)	2.2 ± 1.5^1^	3.9 ± 1.9	4.6 ± 1.5
Mini Mental State Examination	27.18 ± 2.5	27.18 ± 2.5	24.50 ± 2.4
modified Minnesota Impulsive Disorders Interview	0.0 ± 0.0	2.05 ± 4.7	1.00 ± 2.4
39-item Parkinson’s Disease Questionnaire (%)	3.68 ± 2.9	14.07 ± 15.3	17.43 ± 14.3
Non-Motor Symptoms Scale	13.36 ± 12.6^a^	34.86 ± 24.1	27.50 ± 15.4
Tinetti Performance Oriented Mobility Assessment			
Total	27.73 ± 0.5^d^	26.68 ± 1.6^b^	24.83 ± 2.4
Balance subscale	15.73 ± 0.5^d^	15.35 ± 0.9^b^	14.17 ± 1.3
Gait subscale	12.00 ± 0.0^b^	11.32 ± 0.9	10.67 ± 1.2
Functional Gait Assessment	27.91 ± 2.5^d^	25.68 ± 3.0^d^	20.83 ± 4.9
10-m Walking Test (s)	4.50 ± 0.7^d^	5.52 ± 1.2	6.68 ± 2.0
Levodopa currently treated (n, %)	3 (27.3%)	33 (89.2%)	6 (100%)

Characteristics of participants with Parkinson’s disease, grouped by their clinical progress. Data are presented as mean ± SD or n (%). H&Y: Hoehn & Yahr

*Evaluated with the Unified Parkinson's Disease Rating Scale

^a^Significantly different from H&YII (p < 0.05)

^b^Significantly different from H&Y II.5 (p < 0.05)

^c^Significantly different from H&Y II (p < 0.01)

^d^Significantly different from H&Y II.5 (p < 0.01)

All assessments occurred in a dedicated room, free of distractors, and were performed by the same experimenter. The laptop used to run the applications was placed on a table, at a height of 80 cm. The Kinect v2 was placed on the table in front of the laptop, pointing towards the room. The WBB was situated on the floor, 1.5 m away from the table.

### Data analysis

Participants with PD were categorized into three groups, according to their H&Y scores: I–I.5, II, and II.5 [28]. Because only two participants were scored as H&Y I.5, they were merged into the H&Y I group. The mean value of each postural parameter was defined as the mean value of each subtest, to ensure the homogeneity of subjects’ data.

Multiple regression analyses were performed to evaluate the influences of age, sex, education, height, weight, and disease duration on posturographic and gait measurements during the single-task condition. Demographic and clinical differences between groups were investigated using analysis of variance (ANOVA) and frequency analyses, applying Bonferroni and Cramer’s V post hoc corrections to quantitative and qualitative variables, respectively. Student’s t-tests and ANOVA analyses were also used to evaluate differences between all measures of postural control and gait, including (1) between controls and all individuals with PD; (2) between controls and the H&Y I–I.5, II, and II.5 groups, separately; (3) among the H&Y groups; and (4) among groups of participants with different phenotypes. Post hoc Bonferroni correction was applied to all ANOVA tests. The convergent validity of measurements made using instrumented posturography and gait analyses and those made using conventional scales was determined using Pearson’s correlation analyses. The significance level was established at 95% confidence.

## Results

### Participants

Fifty-four individuals with PD (11 individuals in stage I–I.5, 37 individuals in stage II, and 6 individuals in stage II.5) and 43 healthy controls participated in the study.

The three groups of participants with PD demonstrated significantly different scores for subparts II and III of the UPDRS and for the lower body sub-items of part III (Table 1). Significant differences were also found among these groups for disease duration and the scores on the Non-Motor Symptoms Scale, the Tinetti Performance Oriented Mobility Assessment, the Functional Gait Assessment, and the 10-m Walking Test. Other demographic and clinical varia bles showed no significant differences, supporting the existence of inter-group homogeneity. Phenotypes were similarly distributed among groups. Forty-four participants (81.4%) were being treated with levodopa (Table 1).

Twenty-five healthy women (58%) and 18 men (42%), with a mean age of 67.84 years, participated in the study and completed the posturographic and gait assessments. All participants completed all the tests. Data from three healthy participants during the dual-task gait condition could not be retrieved and were not included in the analysis.

### Influence of demographic and clinical variables

Regression analyses showed the statistically significant influences of age on the mean displacement during LOS (β = − 0.461; p = 0.002), gait speed (β = − 0.446; p = 0.002), cadence (β = 0.384; p = 0.030), and stride time (β = 0.438; p = 0.009), of height on gait speed (β = 0.465; p = 0.001) and stride length (β = 0.473; p = 0.002), and of weight on stride width (β = 0.592; p < 0.001).

### Sensitivity to clinical conditions

When the group containing all individuals with PD was analyzed, increased sway was observed compared with the healthy subject group, but no other statistically significant differences in the LOS or gait-related variables were found (Table 2). The in-depth analysis of participants with PD, when grouped by H&Y stage, showed the progressive decline of postural control and gait with increasing H&Y stages. The participants categorized as H&Y II showed the worst performance for all postural and gait measurements among the three groups. In contrast, participants categorized as H&Y I–I.5 not only showed better performance than individuals with more advanced pathological stages but also outperformed healthy subjects, including decreased sway and increased LOS and gait speed values (Table 2).

**Table 2:** Performance of the participants in the instrumented tests of postural control and gait

Postural control	Healthy subjects		Individuals with Parkinson’s Disease							
			All		H&Y I-I.5		H&Y II		H&Y II.5	
Modified clinical test of sensory interaction on balance										
Speed (cm/s)	0.54 ± 0.1		*0.59* ± *0.1*^*a‡*^		0.50 ± 0.1		*0.60* ± *0.1*^*a*^		*0.66* ± *0.1*^a*b‡^	
Limits of stability										
Maximum displacement (cm)	8.65 ± 1.34		8.37 ± 1.6		*9.47* ± *1.5*^*a‡*^		*8.24* ± *1.4*^*b‡*^		*7.14* ± *2.3*^*b*^	
Reaction time (s)	1.09 ± 0.5		1.17 ± 0.5		1.25 ± 0.4		1.05 ± 0.5		*1.75* ± *0.6*^a*c*^	

Gait										
	Single task	Dual task	Single task	Dual task	Single task	Dual task	Single task	Dual task	Single task	Dual task
Gait speed (m/s)	1.1 ± 0.1	1.0 ± 0.2	1.1 ± 0.2	0.9 ± 0.2	1.2 ± 0.1	*1.1* ± *0.2*^*a*^	1.1 ± 0.2	*0.9* ± *0.2*^*b*^	*0.9* ± *0.3*^a‡b*c^	*0.8* ± *0.3*^a*b*^
Cadence (steps/min)	114.8 ± 11.1	103.9 ± 16.4	112.4 ± 13.1	101.1 ± 14.9	*121.4* ± *12.2*^*a*^^**‡**^	108.4 ± 12.2	*112.0* ± *10.7*^*b‡*^	101.3 ± 14.8	*98.8* ± *17.4*^a*b*c^	*88.1* ± *13.0*^*ab*^
Stride length (m)	1.2 ± 0.1	1.1 ± 0.1	1.1 ± 0.1	1.1 ± 0.2	1.2 ± 0.1	1.2 ± 0.2	1.1 ± 0.2	1.1 ± 0.2	*1.1* ± *0.2*^*a*^	*1.0* ± *0.2*^*a‡b‡*^
Stride time (s)	1.0 ± 0.1	1.2 ± 0.2	1.1 ± 0.1	1.2 ± 0.2	1.0 ± 0.1	1.1 ± 0.2	1.1 ± 0.1	1.2 ± 0.2	*1.2* ± *0.2*^a‡b*c^	*1.4* ± *0.2*^a*bc‡^
Stride width (m)	0.1 ± 0.0	0.1 ± 0.0	0.1 ± 0.0	0.1 ± 0.0	0.1 ± 0.0	0.1 ± 0.0	0.1 ± 0.0	0.1 ± 0.0	0.1 ± 0.0	0.1 ± 0.0
Double support time (s)	0.3 ± 0.1	0.3 ± 0.1	*0.3* ± *0.1*^*a‡*^	0.4 ± 0.2	*0.2* ± *0.1*^*a‡*^	0.3 ± 0.1	*0.3* ± *0.1*^*a‡*^	0.4 ± 0.1	*0.4* ± *0.2*^*b*^	*0.5* ± *0.2*^*a*^

Scores of all the participants in the instrumented measures of postural control and gait. Data are presented as mean ± SD. Significant differences after Bonferroni correction are denoted with: only superscript letters for at least 95% confidence; letters plus asterisks * for at least 99% confidence; and letters plus crosses ^‡^ for strong trends (at least 90% confidence)

^a^Different from healthy subjects

^b^Different from the H&Y I-I.5 group

^c^Different from the H&Y II group

The additional cognitive demands required during dual-task execution resulted in deleterious effects for all gait measurements, in both healthy subjects and individuals with PD. Similar to the observed effects on regular walking when participants with PD were considered alone, all gait measurements during the dual-task condition were reduced for higher H&Y stages. However, dual-tasking showed larger differences when groups of participants were compared with healthy subjects, and gait speed differences were detected among the groups of participants (Table 2).

### Sensitivity to phenotype

Individuals with the AR phenotype showed decreased LOS values compared with those for participants with the TD and MX phenotypes. Specifically, individuals with the AR phenotype had lower mean maximum displacement values than those with the TD and MX phenotypes (7.07 vs 8.59 and 8.78 cm, respectively; corrected p < 0.05, in both cases), and greater mean reaction times (1.73 vs 1.07 and 0.98 s, respectively; corrected p < 0.01, in both cases). Although individuals with the AR phenotype showed consistently worse performance for all gait parameters, no other statistically significant differences in postural or gait measurements were found. Similarly, the effect of dual-task conditions on gait performance appeared to be more detrimental for patients with the AR phenotype, but no significant differences were found according to phenotype.

### Convergent validity

Significant correlations were found between the mean displacement and mean reaction times for LOS and the conventional motor scales. No other significant correlations emerged (Table 3).

**Table 3:** Convergent validity between instrumented measures and conventional scales

	Tinetti Performance Oriented Mobility Assessment			Functional Gait Assessment	10-m Walking Test
	Total	Balance	Gait		
Postural control					
Modified clinical test of sensory interaction on balance					
Speed	− .183	− .24	− .088	− .154	.13
Limits of stability					
Maximum displacement	.484**	.413**	.455**	.516**	− .506**
Reaction time	− .354**	− .369**	− 0.265	− .501**	.279*
Gait (single-task)					
Gait speed	.521**	.509**	.422**	.766**	− .850**
Cadence	.447**	.434**	.364**	.594**	− .698**
Stride length	.385**	.360**	.329*	.594**	− .655**
Stride time	− .458**	− .455**	− .363**	− .645**	.750**
Stride width	− .026	.042	.09	− .011	.059
Step length	.293*	.327*	.196	.541**	-.625**
Step time	− .458**	− .414**	− .405**	− .628**	.732**
Double support time	− .475**	− .458**	− .391**	− .592**	.761**

Correlations between measures of the instrumented tests and conventional clinical scales

*p < 0.05. **p < 0.01

All instrumented gait parameters during the single-task condition, except for stride width, showed significant correlations with conventional clinical scales, with variable strengths. Among these correlations, the strongest interactions were found for the 10-m Walking Test, which ranged from good to excellent. Independent of correlation strengths, the signs of the correlations were all coherent, and indicated that better performances on all postural and gait parameters were correlated with better performances on the clinical scales.

## Discussion

In the present study, we investigated the use of the WBB and the Kinect v2 for the comprehensive analysis of posture and gait in PD, focusing on the influence of demographic and clinical variables on postural- and gait-related parameters, the relationships between postural- and gait-related parameters, clinical conditions, and PD phenotype, and the convergent validity of these assessments with clinical scales. We found that postural and gait parameters were differentially influenced by age, height, and weight. Participants with PD showed impaired sway when compared with healthy controls, and a progressive decline in sway was associated with disease worsening. Patients with the AR phenotype had the worst performance among the groups. Different correlations with coherent and variable strengths were found between the posture and gait-related parameters assessed using the instrumented posturography applications and clinical scales.

The effects of demographical variables on posture and gait that were detected in our study are supported by the findings of previous studies, which highlighted age as an unavoidably deleterious factor [40–42], associated with cognitive, muscular, and structural changes. The height-related advantages in gait performance observed during our study have been also reported previously for individuals in similar age ranges as our participants, although this association appears to lose relevance beyond 80 years of age [43]. Similarly, previous studies have identified weight as a factor that influences stride performance [44], which supports the results found in our study.

The increased sway detected during advanced pathological stages has been repeatedly reported [45–47] and is supported by a recent review, which identified this feature as one of the five primary control systems, together with the LOS [48]. Reduced LOS has also been previously described for PD patients [49] and has been associated with an increased risk of falling [50, 51]. The postural assessments performed during our study also demonstrated reduced LOS in participants with PD, which increased with disease progression, but failed to reveal statistical significant differences between PD patients and healthy controls, likely due to the limited number of participants in H&Y stages > II. The differences found during this study between individuals classified as H&Y I–I.5 and those in other stages may be due to the abnormal performance of the H&Y I–I.5 group in this study, which was improved even compared with healthy controls.

Instrumented gait assessment using the Kinect v2 successfully differentiated H&Y II.5-stage PD participants from healthy controls during both single and dual-task conditions for all gait parameters, except stride width. As previously reported [6], walking parameters deteriorated during the dual-task condition in this study. The negative effects of dual-tasking were evident for both healthy controls and individuals with PD, for all gait parameters, except stride width. The absence of changes in stride width during both conditions is supported by a previous study, which reported comparable results among individuals with PD, both with and without cognitive impairments [52]. The additional cognitive demands of the dual-task condition had a larger effect on PD patients in comparison to healthy subjects, which is also consistent with previous reports [53, 54]. The observed enhancement of gait disturbances during the dual-task condition has been explained as a consequence of the activation of parallel processes in frontal attentional areas, which interferes with basal gait commands [55]. Such interference could be less pronounced during the early stages of PD when cognition is generally well-enough preserved to over-compensate for this attentional interference [56, 57]. All these results under the dual-task condition should be taken into account considering that parameters of single-task walking have been reported to be more accurate and have less variability for predicting the progression of the pathology [58].

The performances of H&Y I-I.5 PD patients observed in our study are comparable to those reported in previous studies [22, 59]. Other studies have reported a lack of significant balance or gait alterations during the early stages of PD [3, 59], which may indicate the development of compensatory mechanisms during the early stages of pathology. This abnormal performance could be also explained by the Hawthorne effect, which describes how a subject’s awareness of being observed can motivate an improved performance [60]. Specific analysis of other gait features could have improved the sensitive of the Kinect v2 to the motor differences among early stages of PD. First, reduced arm swing has been identified as a clinical feature of early stages of PD that can be evidenced using instruments, such as inertial sensors [61] and even with the Kinect [62]. Second, dynamic postural control during turning has been also shown to be altered even in the early stages of PD, which can be evidenced with a motion analysis system [63]. Third, subjects in early stages of PD can also have difficulties to maintain a steady gait rhythm, which can manifest as an increased stride-to-stride variability [5, 64]. Future studies should investigate the ability of the Kinect v2 to detect these motor features in early stages of PD, which might be even motor markers of prodromal PD [61]. A few studies have identified differences in gait performance between individuals with the AR phenotype and those with other phenotypes, such as decreased speed and stride compared with those with the TD phenotype [4]. Slower speeds and shorter step lengths have been also described in subjects who present with the freezing of gait, which is more prevalent for the AR phenotype [65, 66], compared with healthy controls [5]. Our study suggests that patients with the AR phenotype show slightly more impaired balance and increased gait disturbances compared with patients with other phenotypes. The limited number of participants with an AR phenotype included in this study may have impaired the ability to observe significant differences among phenotypes.

The convergent validity of the instrumented postural and gait assessments with conventional clinical tools in this study was comparable not only to that of the clinical tools used [67] but also to that of laboratory-grade and gold-standard systems [68, 69]. More importantly, the assessments made by the instrumented tests were consistent (that is, had the same correlation sign) with those made using clinical scales. For instance, increased sway was associated with an increased time necessary to perform the 10-m Walking Test, and lower scores in the Tinetti Performance Oriented Mobility Assessment and the Functional Gait Assessment.

The ability detect the dependence of posture and gait performance on demographic variables, the sensitivity to discriminate between healthy controls and individuals during the middle stages of pathology, and the convergent validity with clinical scales support the usefulness of these low-cost instrumented tools as complements to the clinical assessment of postural control and gait in PD patients. These results, together with the low-cost and portability of the tested devices, could also support their use as a low-cost alternative to laboratory-grade systems in the clinical setting, to complement postural and gait assessments. Despite their widespread availability, it is important to consider that these devices have been discontinued and, consequently, future studies should investigate the feasibility of using the latest generation of low-cost cameras, such as the Azure Kinect (Microsoft, Redmond, WA, USA), and deep learning algorithms of computer vision to assess human posture and gait.

## Conclusions

In summary, the instrumented assessment of postural control and gait in individuals with PD, using low-cost devices, may provide feasible and complementary information to conventional measures that are used to diagnose and monitor pathological progression.

## Data Availability

The datasets used and/or analysed during the current study are available from the corresponding author on reasonable request.
